# The Influence of CTAB-Capped Seeds and Their Aging Time on the Morphologies of Silver Nanoparticles

**DOI:** 10.1186/s11671-019-2898-x

**Published:** 2019-03-05

**Authors:** Wenxiu Jin, Guorun Liang, Yuanzhi Zhong, Yongcong Yuan, Zhichao Jian, Zhixiong Wu, Wanzhong Zhang

**Affiliations:** Guangdong Provincial Key Laboratory of New Drug Screening, School of Pharmaceutical Sciences, Southern Medical University, Guangzhou, 510515 People’s Republic of China

**Keywords:** Silver nanoparticles, Size and morphology control, Seed crystals, Aging time, Cetyltrimethylammonium bromide

## Abstract

Contrast to the polydisperse nanorods formed by common seed-mediated growth method without the presence of cetyltrimethylammonium bromide (CTAB) in seed solution, we successfully obtained silver nanoparticles with different morphologies in the same reaction system by addition of CTAB in the seed solution. In this work, an appropriate amount of CTAB was added into the solution to prepare silver seed crystals. The results show that the aging time of silver seeds have a great influence on the sizes and morphologies of silver nanoparticles and thus the shape-controllable silver nanoparticles can be easily achieved by simply changing the seed aging time. The results also support that the selective adsorption ability or adsorption behavior of TSC can be adjusted by adding CTAB in the preparation procedure of silver seeds. We suggest that different aging times generate different effects on the competitive adsorption between CTAB and citrate to induce the orientation growth of silver seeds. As a result, silver nanospheres, nanorods, and triangular nanoplates can be easily prepared in the same system. In addition, we overcome the time limitation about the use of the seeds by adding CTAB into seed solution and make the synthesis of silver or other metal nanoparticles with different morphologies more easily and more efficiently.

**Electronic supplementary material:**

The online version of this article (10.1186/s11671-019-2898-x) contains supplementary material, which is available to authorized users.

## Background

Silver nanoparticles (AgNPs), a noble metal nanostructure, have always been a hot research topic over the years. Due to their surface effect [1], quantum size effect [2], macroscopic quantum tunneling effect [3], and other unique properties, AgNPs have been used in many fields successfully [4]. For instance, AgNPs can be applied as antimicrobial materials [5–7], anticancer materials [8], catalytic materials [9, 10], DNA detection materials [11], and drug delivery carriers [12]. Research results show that the physical and chemical properties of anisotropic nanoparticles, such as nanorods, nanowires, and nanoplates, are strongly influenced by their particle sizes [13] and morphology [14, 15]. Therefore, the study on the size-controlled and morphology-controlled synthesis of AgNPs is important and challenging now.

Initially, silver nanoparticles were synthesized by various routes, including lithographic techniques, biological techniques, physical methods, and chemical methods [16–18]. Among those, wet chemical reduction method has been a distinguished one since it is simple to fabricate uniform particles and suitable to be applied in large-scale production. As for the development of wet chemical synthesis, many researchers devoted themselves. Xia et al. used polyvinyl pyrrolidone (PVP) as reaction medium and prepared high-quality nanowires [19]. Mirkin’s group first presented synthesis of triangular nanoparticles in liquid phase with optical radiation, and their experiments elucidated the optical characteristics of nanoprisms and nanoplates [20].

A seed-mediated method is convenient to control the size and morphology of the resulting nanoparticles [21–23]. It has developed rapidly nowadays, and the study of growth mechanism is progressing. However, there are still many unclear factors waiting to be addressed. Murphy et al. firstly introduced the seed-mediated method in 2001 [24], which had a profound impact on researchers followed. In general, the growth of anisotropic nanoparticles occurred in the presence of cetyltrimethylammonium bromide (CTAB) and the size of formed nanorods was controllable in the colloidal solution. However, a large number of spherical particles mixed in products and the products needed several separations, resulting in low yield. In addition, the initial method proposed by Murphy noted that the seeds should be used in a limited time interval. There is not any good solution for solving time-limited problem all the time, and the research progress of the seed-mediated method is still limited. Research showed that CTAB-capped seeds were used instead of a citrate-capped one to prepare gold nanorods, making the regularity of obtained particles markedly improved [25]. The result indicated that CTAB played a critical role in the growth of seed crystals. CTAB molecules have a high affinity for the (110) facet and induce anisotropic growth of the seeds. This may be a major factor in improving the regularity of nanoparticles.

Because of high surface energy, single nanoparticles usually form aggregations easily. The addition of special protective agent can make the surface inactive and prevent the formation of nanoparticle aggregations. CTAB, a popular surfactant, can form micelles when its concentration is over the critical micelle concentration (CMC). Besides, the selective adsorption of CTAB on the surface induces the orientation growth of the seed crystals.

In this paper, we used an improved seed-mediated method to synthesize silver nanoparticles with different morphologies. When preparing silver seed crystals, we added particular concentration of CTAB to adjust the selective adsorption on the surface of the seed crystals, and thus, it would induce the anisotropic growth of the seed crystals. Based on this method, we prepared nanospheres, nanorods, and nanoplates in the same system and the only different factor was the aging time of silver seeds. In addition, our seeds can be used from the beginning to about 52 h and more. As a result, we overcome the limitation about the seeds and make the synthesis of silver or other metal nanoparticles with different morphologies more easily and more efficiently.

## Methods

In order to investigate the influence of CTAB-capped seeds and their aging time on the morphologies of silver nanoparticles, an appropriate amount of CTAB was added into the solution to prepare silver seed crystals. Then, these seeds aged for different times were used to prepare AgNPs with different morphologies.

### Materials

Silver nitrate (AgNO_3_), potassium borohydride (KBH_4_), sodium hydroxide (NaOH), trisodium citrate (TSC), and ascorbic acid (V_c_) were all analytically pure (AR) and used without further purification. Cetyltrimethylammonium bromide (CTAB) was purchased from AMRESCO LLC. Water used in the experiments was double distilled.

### Instruments

The particle size distribution of silver seeds was determined by Zetasizer Nano ZS90 (Malvern Instruments, Malvern, UK) in the dynamic light scattering (DLS) regime for particle size distribution, equipped with an avalanche photodiode for signal detection. The concentration of seed solution was diluted to one tenth with double distilled water when measuring. U-3900 UV-vis spectrophotometer recorded the resonance absorption of the formed silver nanoparticles. Transmission electron microscope (TEM) images were acquired on a JEM-1400 transmission electron microscope.

### Preparation of Silver Seeds

0.2 mL of 0.1 M CTAB, 0.5 mL of 0.01 M AgNO_3_, and 0.5 mL of 0.01 M TSC were added in 19.0 mL distilled water in order. Next, 0.6 mL of 0.01 M freshly prepared ice-cold KBH_4_ was added into the reaction solution rapidly at once. Then, the reaction solution was stirred gently. It was better to keep the reaction system at 28 °C. The solution turned bright yellow, implying the formation of silver nanocrystals. About 10 min later, the solution turned yellow-green. The nanocrystals in this solution aged for different time were used as seeds from the beginning to 52 h, even longer. In contrast, the seeds were prepared by adding CTAB but without adding TSC, and other conditions were the same as the above case.

### Preparation of Silver Nanoparticles

In a 50-mL clean and dry conical flask, 15.0 mL of 0.1 M CTAB and 0.5 mL of 0.01 M AgNO_3_ were added. Next, 0.25 mL seed collosol prepared and aged for different times was added into the mixture solution. Then, 1.0 mL 0.1 M V_c_ and 3.0 mL 0.1 M NaOH were added, and the solution was stirred quickly and acutely for 3 min. The solution turned deep-yellow, brownish red, and blue-black, which corresponds the aging time of the seeds. As a comparison, the color of AgNPs colloidal solution was yellow and did not change with the aging time of the silver seeds prepared by adding CTAB but without adding TSC.

## Results and Discussion

### Formation of Silver Nanoparticles by the Seeds at Different Aging Time

Silver nanorods have two typical absorption peaks, i.e., the transverse plasmon band (centered at ~ 400 nm) and longitudinal plasmon band [26, 27]. Triangular silver nanoparticles have three characteristic absorption peaks derived from their in-plane dipole plasmon resonance, in-plane quadrupole resonance, and out-of-plane quadrupole resonance [20].

UV-vis spectra in Fig. 5a showed the spectral absorption of silver nanoparticles generated by the seeds at different aging time. From the spectral change tendency, it is observed that the seeds aged for different time have a big effect on the morphology of the formed AgNPs. The obtained nanoparticles prepared by the fresh seeds have only one principal plasmon band at ~ 412 nm, indicating that the formed nanoparticles are almost nanospheres. While the nanocrystals aged for 10 min are used as seeds, there is a new but small absorption peak appeared at 480 nm, indicating that silver nanorods are beginning to form. However, the absorption peak at ~ 412 nm is higher than the one at ~ 480 nm, which is probably caused by lots of spherical nanoparticles mixed in the product. Then, by using the seeds aged for 15 min, a shoulder peak at ~ 345 nm becomes more and more obvious. By using the seeds aged over 15 min, the peak intensity at ~ 412 nm becomes lower and the maximum absorption wavelength (*λ*_max_) has a red shift while the peak intensity at ~ 500 nm becomes higher. While the seeds are aged for about 30 min, it can be seen a typical resonance absorption of triangular nanoparticles in the UV-vis spectrum. From the spectral change tendency, the absorption peak centered at ~ 412 nm continuously decreases and the peak centered at ~ 500 nm gradually rises with an obvious red shift. At ~ 350 nm, it is first a shoulder peak and then a small peak finally. These spectral phenomena imply that the morphology of the formed nanoparticles changes significantly in the use of the seeds aged within the first 30 min.

**Fig. 1:**
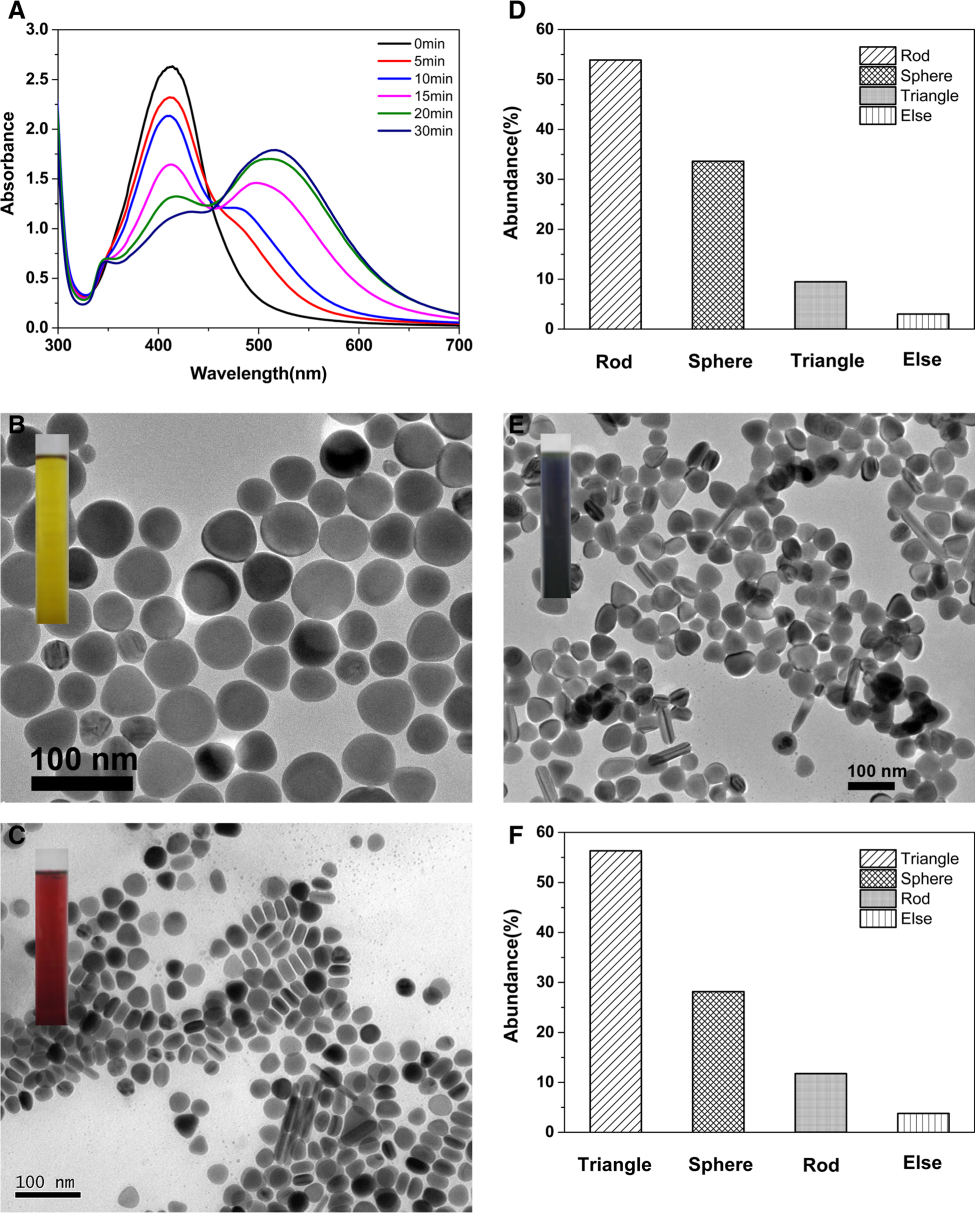
**a** UV-vis spectra of nanoparticles obtained at different seed aging times. **b**, **c**, **e** TEM images of silver nanospheres prepared by the seeds aged for 0 min, silver nanorods prepared by the seeds aged for 15 min, and silver triangular nanoplates prepared by the seeds aged for 30 min. **d**, **f** Shape-distribution histograms of AgNPs corresponding to the TEM images of **c** and **e**; the statistical numbers of the particles are 279 and 308, respectively

TEM images in Fig. 1b, c, and e showed the morphologies of obtained nanoparticles prepared at different seed aging times. The lower magnification TEM images of silver nanorods and triangular nanoplates corresponding to Fig. 1c, e were presented in Additional file 1: Figure S4. It is observed that the obtained AgNPs are corresponding with the deductions from the above resonance absorption. The shape-distribution histograms shown in Fig. 1d and f indicate that the morphologies of the main nanoparticles change from the nanospheres to nanorods and to triangular nanoplates, while the AgNPs are prepared by our seeds that aged for different times from 0 to 30 min. While the fresh seeds were used (that is, the seeds were not aged), silver colloidal solution presented deep yellow in color (the inset image in Fig. 1b). The formed nanoparticles shown in Fig. 1b were mainly silver nanospheres and near nanospheres with av. diameter of about 41.0 ± 14.3 nm. Some truncated nanotriangles also intermixed in the nanospheres and near nanospheres (the shape-distribution histograms of AgNPs is not presented).

**Fig. 2:**
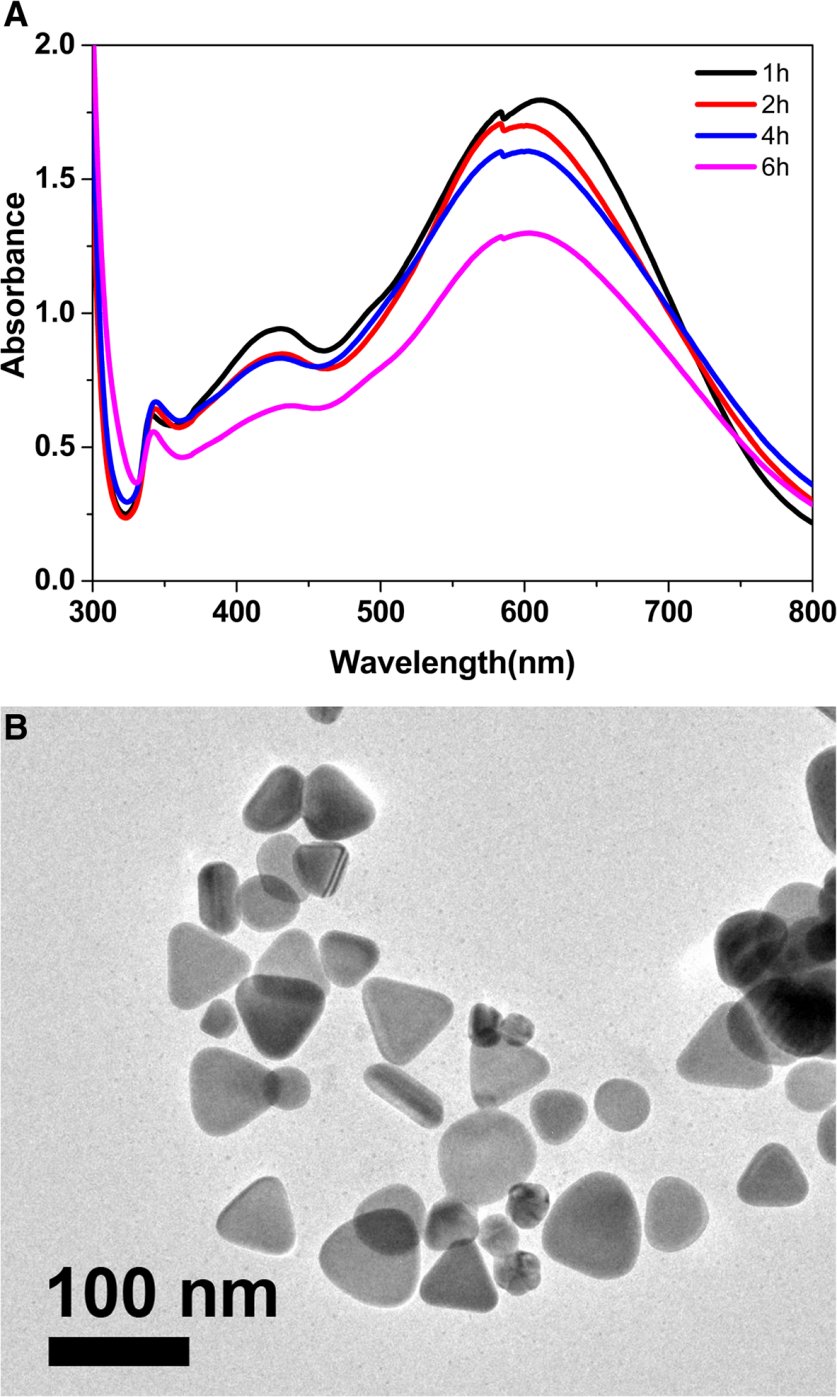
**a** UV-vis spectra of the obtained nanoparticles prepared by the long-aged seeds. **b** TEM image of the truncated triangular nanoplates prepared by the seeds aged for 6 h

When the seeds were aged for 15 min, the formed nanoparticles shown in Fig. 1c were mainly silver nanorods and the colloidal solution presented brownish red in color (the inset image in Fig. 1c). In addition, there are some spheroidal and a few triangular nanoparticles appeared as associated by-products of the nanorods. The shape-distribution histograms of the formed AgNPs shown in Fig. 1d implied that the abundance of silver nanorods attained to about 53.9% and the abundance of the main associated nanoparticles, i.e., silver nanospheres, was about 33.6%. While the seeds were aged for 30 min, the formed nanoparticles shown in Fig. 1e were mainly triangular nanoplates and silver colloidal solution presented blue-black in color (the inset image in Fig. 1e). The obtained triangular nanoparticles are truncated in shape. Fig. 1f showed that the abundance of silver triangular nanoplates, nanospheres, and nanorods attained to about 56.3%, 28.2%, and 11.8%, respectively.

It had been thought that the seeds required to age for at least 2 h after preparation, and after 5 h, there was a thin film of nanoparticles appeared at the surface of seed solution, indicating the nanocrystal aggregation. Thus, the seeds could be used 2 h but could not be used 5 h after preparation [24]. As for the reason why new seeds should be aged for some time before use, no further explanation is noted in their study. We suppose that the seed crystals were not formed well, and there were crystal defects just after the preparation of the seeds. The seeds aged for a suitable time (e.g., 2 h) help the selective adsorption of surfactant molecules on the special crystal surface. The seeds aged for a long time (e.g., 5 h) result in the all-sided adsorption of surfactants on the seed crystals and the formation of complete crystalline nanoparticles as well as the aggregation of the seed nanocrystals.

Figure 2a showed the UV-vis spectra of obtained nanoparticles prepared by the seeds that were aged for a long time. The absorption peaks at ~ 600 nm, 420 nm, and 350 nm do not change obviously in the maximum absorption wavelength, but the absorption intensity decrease, implying that the obtained nanoplates decrease with the aging time extended. Figure 2b showed TEM image of nanoplates prepared by the seeds that were aged 6 h. It indicates that the obtained nanoparticles prepared by the seeds, which are aged for a long time, are almost triangular nanoplates with the av. side length of about 52.2 ± 10.3 nm. The obtained triangular nanoplates are also truncated in shape, and some nanospheres are mixed among them because of the competitive growths between the unadsorbed and adsorbed lattice planes of silver seeds. As a result, the seeds prepared by our improved seed-mediated method are different from the published study and our seeds can be used from just being prepared to a quite long time by addition of appropriate CTAB in the seed preparation.

### How Does CTAB Added into the Seed Solution Affect the Formation of AgNPs?

Trisodium citrate (TSC) is an important chemical in the preparation of silver seeds to determine the morphology of the formed nanoparticles [28]. How does CTAB added into the seed solution affect the formation of AgNPs? What will happen if CTAB instead of TSC is added in the procedure of silver seeds? It has not been reported in the published literatures. In order to study the influence of CTAB and TSC in the preparation procedure of silver seeds, the contrast experiments were carried out using silver seeds with and without TSC in the preparation procedure.

**Fig. 3:**
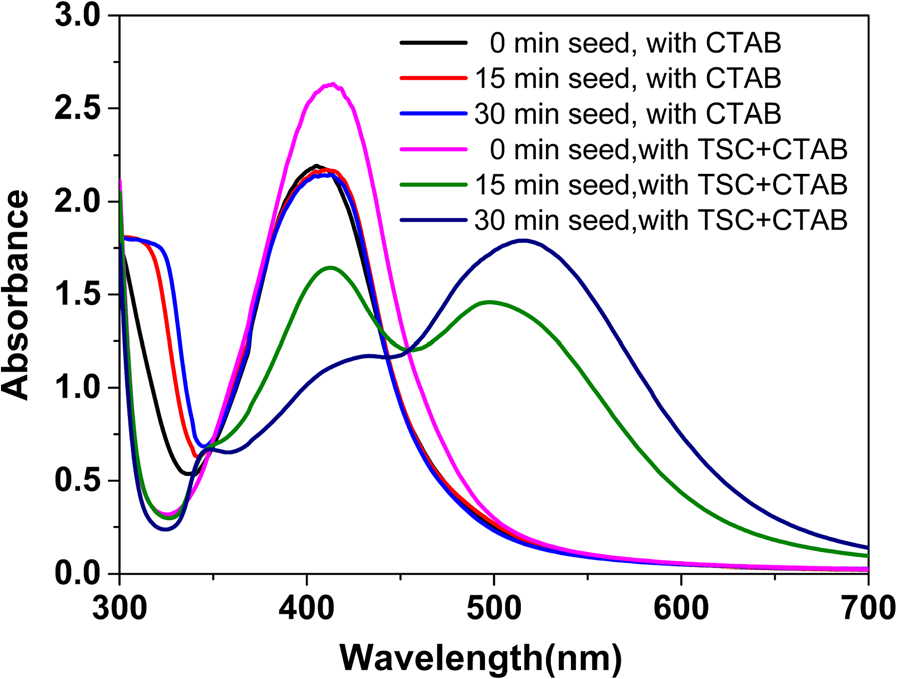
UV-vis spectra of the AgNPs prepared by using the two types of silver seeds (with or without adding TSC) at different aging times

The UV-vis spectra shown in Fig. 3 showed the formation of silver nanoparticles by using the two silver seeds above (with or without adding TSC) at different aging times. Obviously, silver nanospheres, nanorods, and triangular nanoplates had formed by the silver seeds that were aged for 0, 15, 30 min in our reaction system (adding both TSC and CTAB). These results are in good agreement with the previous experimental studies (“Formation of Silver Nanoparticles by the Seeds at Different Aging Time” section). In contrast, the color of AgNPs colloidal solution was yellow and did not change with the aging time of the silver seeds extended (0 ~ 30 min), when TSC was absent in the preparation procedure. Besides, it can be seen the characteristic absorption (centered at ~ 400 nm) of silver nanospheres in the UV-vis spectra, indicating that only silver nanospheres were formed by using silver seeds (with CTAB and without TSC) that were aged for 0, 15, and 30 min. The above experimental results showed that the seeds prepared by just adding CTAB had grown to spherical nanoparticles, implying that the growths of the seed crystals were nonselective, that is, the adsorption of CTAB molecules on the crystal planes of silver seed crystals has no selectivity.

However, the results support that the selective adsorption ability or adsorption behavior of TSC can be adjusted by adding CTAB in the preparation procedure of silver seeds (see our experimental results about adding both TSC and CTAB in Fig. 3). Furthermore, the aging time of the seed collosol has a big influence on the selective adsorption behavior derived from the new seeds in our case. As a result, the morphology and particle size of the formed nanoparticles can be controlled through the following ways: (1) by changing in the aging time of the silver seeds prepared by adding both TSC and CTAB and (2) by adjusting the addition of TSC and CTAB in the procedure of silver seeds [29].

It is obvious that the influence of CTAB in seed solution is significant in controlling the nanoparticles’ morphology and size. Here, we carry out theoretical calculations and experimental study to verify the effect of CTAB in seed solution. At 30 °C, the first CMC of CTAB is 0.72 mM and the second CMC is 9.6 mM. If the concentration of CTAB is between first CMC and second CMC, the formed micelles are spherical. While the concentration of CTAB is higher than its second CMC, the micelles change from spherical to rod-like ones [30]. In our experiment, the concentration of CTAB in the seed solution is 0.96 mM. Apparently, CTAB forms spherical micelles in seed solution.

In the theoretical calculation, it can be confirmed that precipitation reaction between Ag^+^ and Br^−^ is dominant in the system, indicating that most of Ag^+^ reacts with Br^−^ instead of citrate [29]. It can slow down the reducing procedure and thus reduce the concentration of free Ag^+^. The formed AgBr are rapidly reduced to Ag with the addition of KBH_4_. Then, the large amounts of Ag atoms are absorbed into spherical micelles, avoiding the conglomeration among the mini silver nanoparticles. However, AgBr precipitates produced by the reaction between AgNO_3_ and CTAB may decompose under light. The formation of silver seeds or AgNPs may derive from a competition between the decomposition and reduction of AgBr. In order to study the competition of the decomposition and reduction, the contrast reaction for the preparation of AgNPs was carried out by with and without adding NaOH in the system (Additional file 1: Figure S1). The results showed that the reaction solution was still a colorless transparent solution and no obvious absorption peaks were observed within 60 min, implying that the AgBr precipitate in this system did not decompose or the decomposition rate of AgBr was negligible under light.

The reduction rate of silver ions is controlled to a high degree by the acidity-basicity of the V_c_ reaction solution [31]. The ionization of V_c_ depends on the acidity-basicity of the solution, and the redox potential of silver ions is influenced by the difference in complexing action between silver ion with the monoanion and dianion of V_c_. For the formation of AgNPs by adding NaOH, only 3 min was needed to carry out the synthesis of silver triangular nanoplates and nanorods or near nanospheres. On the contrary, silver ions are not reduced by V_c_ in the solution without NaOH. For the formation of silver seeds by adding CTAB and TSC in our system, the experimental results are similar to that obtained from the above experiments (Additional file 1: Figure S2). That is, the AgBr precipitate in the preparation of both silver seeds and AgNPs do not decompose or the decomposition rate of AgBr is negligible under natural light in our system. The stability in the photodegradation of AgBr should derive from the AgBr precipitate capped by CTAB micelles or adsorbed by CTAB and citrate competitively in our system.

**Fig. 4:**
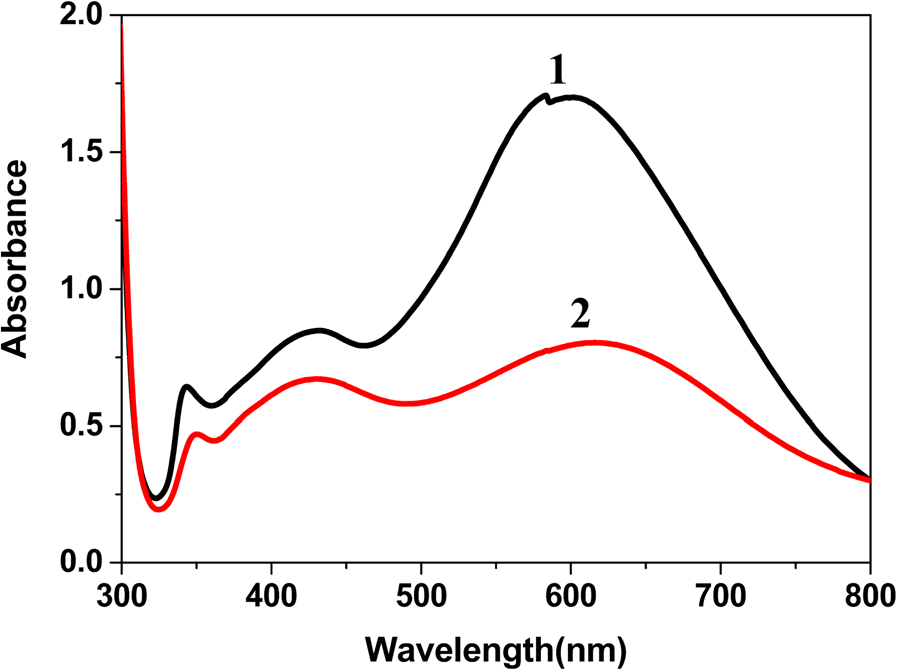
UV-vis spectra of AgNPs obtained from two different seeds prepared by using 0.1 M CTAB (1) and 0.1 M NaBr (2) respectively and aged for the same time (20 min)

In order to further study the crucial role of CTAB, we prepared two different seed crystals by using 0.1 M NaBr and 0.1 M CTAB, respectively. Figure 4 is UV-vis spectra of silver nanoparticles prepared by the above two seeds. The spectra of AgNPs (by using 0.1 M NaBr) do not change obviously in the maximum absorption wavelength. However, its absorption intensity decreases remarkably. The absorption peak at the direction of longer wavelength (centered at ~ 600 nm) has a lower optical intensity. It implies that the formed nanoparticles are polydisperse in the seed solution. Related researches exhibited that Br^−^ can strongly bond to Ag^+^ to form AgBr that inhibits the growth of silver seeds [29, 32]. According to our experimental results, it explains that CTAB has two main functions in the formation of silver seeds, i.e., bonding to silver to form AgBr to decrease the reduction rate of Ag^+^ and showing its selective adsorption in the presence of TSC to induce the orientation growth of silver seeds.

### What Happened of the Seeds on Their Aging Procedure?

Some researchers suggest that the aging has an influence on only small nanocrystals [33]. Researches related to the seed aging time showed that the seeds should be used in a limited time interval after preparation. In the study, we overcome the disadvantage and can produce various silver nanoparticles in a simple system. Herein, we attempt to find out what happened of the seeds on their aging procedure.

**Fig. 5:**
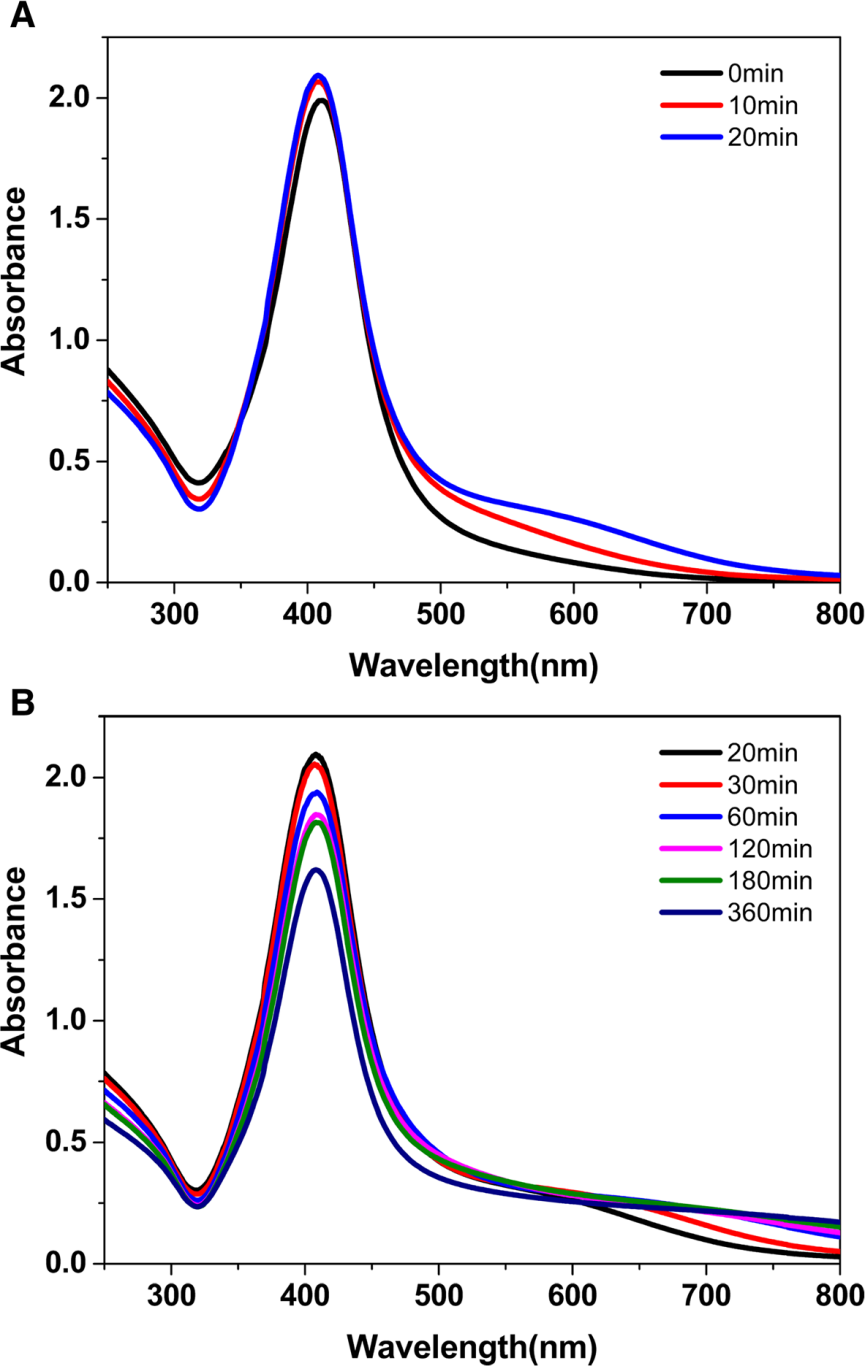
UV-vis spectra of the seed crystals that were aged from 0 to 6 h, **a** 0–20 min. **b** 20–360 min

UV-vis spectra in Fig. 5 showed the absorption changes of the seed crystals during the period of the seeds aging from 0 to 6 h. Only one principal plasmon peak at ~ 400 nm indicated that the formed seed crystals were nanospheres, which were the same as the morphology of silver seeds prepared with just TSC [34]. The maximum absorption wavelengths (*λ*_max_) are 411, 410, 408, 409, 409, 408, 408, and 408 nm with the corresponding seed aging time 0, 10, 20, 30, 60, 120, 180, and 360 min respectively. From 0 to 20 min, the *λ*_max_ has a blue shift of 3 nm (as shown in Fig. 5a). After 20 min, the *λ*_max_ nearly has no change, but full width at half maximum (FWHM) of resonance absorption of the seed collosol decreases gradually with the aged time (as shown in Fig. 5b). The absorption band is narrower with a decrease of FWHM, and we can predict that the particle size increases [35]. From the spectra in Fig. 5b, there is a decrease in the absorption intensity, which may be caused by the formation of a thin film of particles to decrease the amount of silver seeds in colloidal solution. The result is in agreement with that in the published literature [24]. However, the aging time did not affect the use of the seed solution in our experiments, even if the seed solution was aged for more than 6 h.

As shown in Fig. 5, the absorption of the seed solution in the long longitudinal surface plasmon resonance (over 600 nm) increases with the aging time. When the seeds’ aging time is from 0 to 60 min, the absorption over 600 nm increases gradually. Because the seed collosol prepared by citrate without CTAB nearly has no absorption over 600 nm [33], we suggest that the appearance in the absorption over 600 nm reflects the change in the surface-state charge density of the seeds. In our system, both TSC and CTAB are able to adsorb on the crystal face of silver seeds. Due to the opposite electrical properties, we speculated that the surface-state charge density changed with the aging time of silver seeds by the competitive selective adsorption of CTAB and citrate on the surface of the seeds. As a result, silver nanoparticles with different morphologies can be prepared by the seeds aged for different times. At 0 min, there is no adsorption and thus the growth of silver nanoparticles prepared by the fresh seeds show no anisotropy. As a result, the obtained nanoparticles are nanospheres and show a typical absorption at ~ 410 nm. With a short seed aging time (e.g., 15 min), the competitive adsorption of citrate to the seeds is dominant (the absorption over 600 nm is weak). In this case, the anisotropic growth of silver seeds occurred under the guidance of rod-like micellar templates formed by CTAB to form silver nanorods. With a long seed aging time (e.g., over 30 min), the competitive adsorption of CTAB is dominant (the absorption over 600 nm is obvious). When the seeds were aged for over 60 min, the competitive adsorption between citrate and CTAB achieves a balance and the absorption over 600 nm has a maximum and remains unchanged.

While the fresh seeds or the seeds aged for a short time were used, the unreacted BH_4_^−^ in the seed solution might have some impact on the formation of AgNPs. As shown in Additional file 1: Figure S3, it is clear that the change in the quantity of KBH_4_ has little influence on the formation of silver seeds and AgNPs prepared by the seeds. That is to say, the unreacted BH_4_^−^ is not a key factor to determine the morphologies of the formed nanoparticles. The detailed experimental results and explanation can be seen in the section 2 of Additional file.

**Fig. 6:**
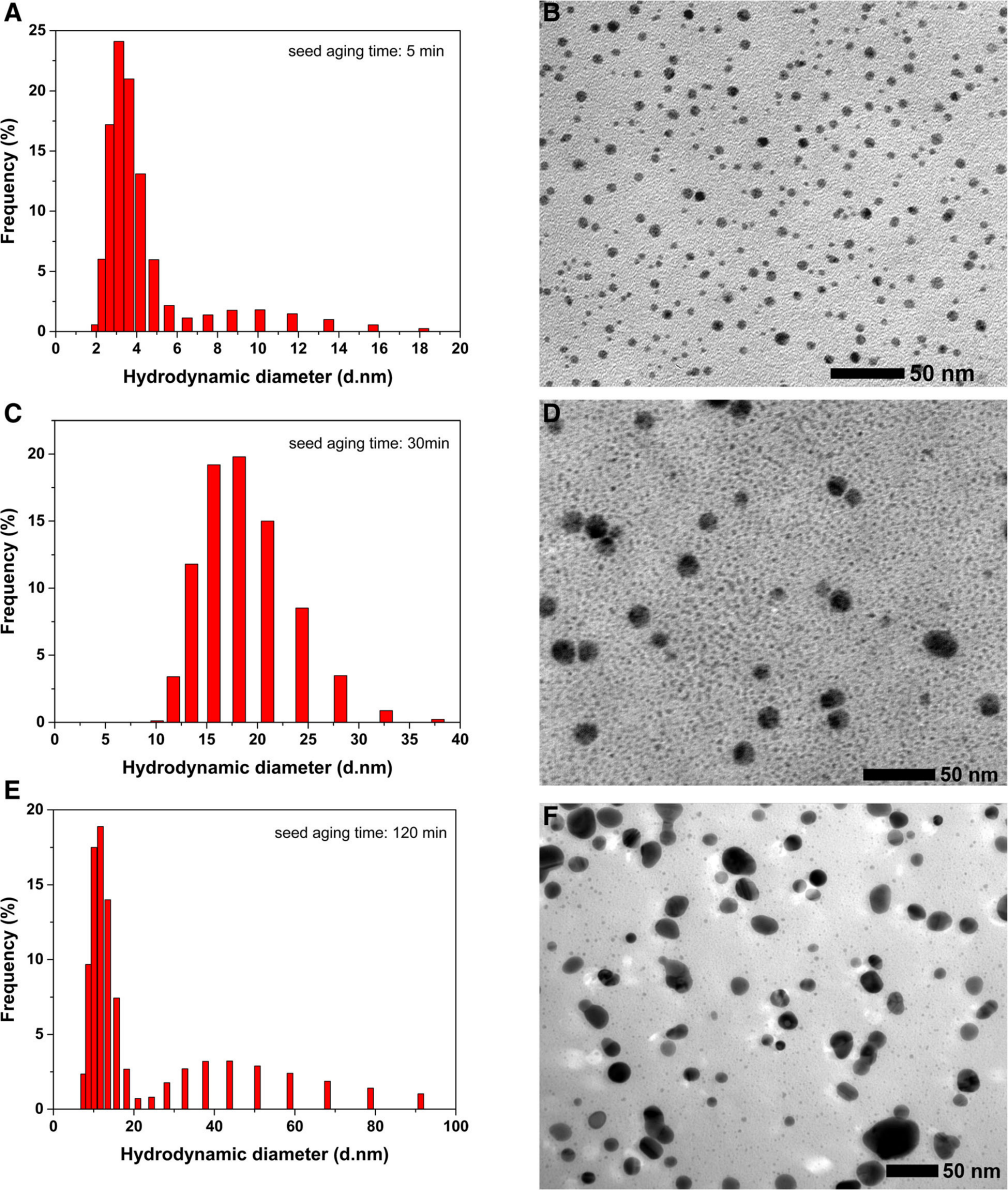
Hydrodynamic diameter distributions of silver seeds characterized via DLS and the corresponding TEM images at different aging times: **a**, **b** 5 min. **c**, **d** 30 min. **e**, **f** 120 min

Figure 6 showed the hydrodynamic diameter distributions of silver seeds at different aging times. The hydrodynamic diameter was characterized via DLS. As shown in Fig. 6a, c, and e, the average hydrodynamic diameters of silver seeds in the aging procedure at 5 min, 30 min, and 120 min are 3.77 ± 0.2 nm, 15.09 ± 0.2 nm, and 17.54 ± 0.2 nm. The hydrodynamic diameter of the seeds is getting larger and larger with the time in the aging procedure. Their corresponding TEM images were presented in Fig. 6. It is clear that the seed crystals are all spherical nanoparticles and their particle size increases with the seed aging time. As shown in Fig. 6b, the formed seed crystals that aged for 5 min are very small and their av. particle size is about 4.9 ± 1.6 nm, which is roughly identical to the hydrodynamic diameter via DLS. Figure 6d showed that the formed seed crystals that aged for 30 min were some bigger spherical nanoparticles with the av. particle size of 16.0 ± 3.0 nm. While the silver seed collosol was aged for a longer time, e.g., 120 min, there was a degree of aggregation between the seed crystals, as shown in Fig. 6f. The size of small part of seed crystals increases to more than 20 nm, and their av. particle size is about 16.9 ± 7.3 nm. These direct data showed the tendency to enlarge the particle size of the seeds with their aging time, which were corresponding with the results derived from the hydrodynamic diameter and the deduction from the UV-vis spectral changes.

It was reported that the vertex growth of triangular nanoparticles was controlled by (111) facet and the lateral growth is controlled by (100) facet [36]. Citrate has a favorite adhesion to Ag (111) facet [37–40] and inhibits the growth of this facet [41]. In our case, Br^−^ derived from CTAB was added in the seed solution to form AgBr with Ag^+^, which affects the relative growth ratio of (111, 100) facet of silver seeds. In addition, the competitive adsorption between citrate and CTAB achieves a balance on the seed surface to further adjust the relative growth ratio of the (111, 100) facet. As a result, the seeds can controllably grow to form truncated triangular nanoparticles. That is to say, we can obtain nanoparticles with different morphologies in the same reaction system by controlling the aging times of silver seeds.

## Conclusions

By using an improved seed-mediated method, we successfully obtained silver nanoparticles with different morphologies in the same reaction system. With the addition of CTAB in seed solution, we can achieve shape-controllable goal for silver nanoparticles by only simply changing the seed aging time. The seed collosol prepared by this method is very stable and can be used from 0 to 6 h and more. The seeds can be used immediately to form silver nanospheres. Silver nanorods and truncated triangular nanoplates can be prepared respectively by using the seeds aged for different times. The aging time of silver seeds is a key factor to form AgNPs with different morphologies.

Contrast to the polydisperse nanorods formed without the existence of CTAB in the seed solution, triangular nanoplates were easily prepared by the seeds added CTAB in moderation and aged for an appropriate time. The size of silver seeds nanocrystals increases with the aging time. We suggest that different aging times generate different effects on the competitive adsorption between CTAB and citrate. Thus, the nanospheres will be formed by the fresh seeds and the nanorods will be formed by the seeds aged for a shorter time (that is, the selective adsorption of citrate to the seeds is dominant). Similarly, triangular nanoplates can form by the seeds aged for a longer time (that is, the selective adsorption of citrate to the seeds is obviously adjusted by CTAB). These results imply that the adsorption balance of CTAB and citrate can affect the growth rate on different crystal faces to induce the orientation growth of silver seeds to form AgNPs with different morphologies, although the detailed mechanism is not that clear now.

## Additional file

Additional file 1: **Figure S1.** UV-vis spectra of the AgNPs prepared by the normal reaction with NaOH in 3 min and the contrast reactions without NaOH in different reaction times respectively: (A) the seeds were aged for 20 min; (B) the seeds were aged for 10 min. **Figure S2.** UV-vis spectra of silver seeds prepared without KBH_4_ and aged at different times. **Figure S3.** UV-vis spectra of the silver seeds prepared by adding different quantity of BH_4_^−^ and corresponding AgNPs: silver seeds aged for 5 min (A) and 15 min (C); AgNPs prepared by the seeds aged for 5 min (B) and 15 min (D). **Figure S4.** Low magnification TEM images of silver nanorods (left) and triangular nanoplates (right) corresponding to Fig.1 (C) and (E). (DOCX 1590 kb)

## Abbreviations

AgNPs: Silver nanoparticles
CMC: Critical micelle concentration
CTAB: Cetyltrimethylammonium bromide
DLS: Dynamic light scattering
FWHM: Full width at half maximum
PVP: Polyvinyl pyrrolidone
TEM: Transmission electron microscope
TSC: Trisodium citrate
V_c_: Ascorbic acid
λ_max_: Maximum absorption wavelength
