# Supplementary material for: The Influence of CTAB-Capped Seeds and Their Aging Time on the Morphologies of Silver Nanoparticles
Source: Nanoscale Res Lett. 2019 Mar 5;14:81. doi: 10.1186/s11671-019-2898-x (PMC6401074; doi:10.1186/s11671-019-2898-x)
Supplement: Supplementary file 1 — Figure S1. UV-vis spectra of the AgNPs prepared by the normal reaction with NaOH in 3 min and the contrast reactions without NaOH in different reaction times respectively: (A) the seeds were aged for 20 min; (B) the seeds were aged for 10 min. Figure S2. UV-vis spectra of silver seeds prepared without KBH4 and aged at different times. Figure S3. UV-vis spectra of the silver seeds prepared by adding different quantity of BH4− and corresponding AgNPs: silver seeds aged for 5 min (A) and 15 min (C); AgNPs prepared by the seeds aged for 5 min (B) and 15 min (D). Figure S4. Low magnification TEM images of silver nanorods (left) and triangular nanoplates (right) corresponding to Fig.1 (C) and (E). (DOCX 1590 kb) [file 11671_2019_2898_MOESM1_ESM.docx]

**Supplementary Materials**

**The influence of CTAB-capped seeds and their aging time on the morphologies of silver nanoparticles**

Wenxiu Jin^a, ‡^, Guorun Liang^a, ‡^, Yuanzhi Zhong^a^, Yongcong Yuan^a^, Zhichao Jian^a^, Zhixiong Wu^a^, Wanzhong Zhang^a,^ *

^a^ Guangdong Provincial Key Laboratory of New Drug Screening, School of Pharmaceutical Sciences, Southern Medical University, Guangzhou 510515, P R China

1. The influence of natural light on the formation of AgNPs without NaOH and silver seeds without KBH_4_

1) The effects of natural light on the formation of silver nanoparticles prepared with NaOH and without NaOH respectively

**B)**

**A)**

Fig. S1 UV-vis spectra of the AgNPs prepared by the normal reaction with NaOH in 3 min and the contrast reactions without NaOH in different reaction times respectively: (A) the seeds were aged for 20 min; (B) the seeds were aged for 10 min.

In order to study the competition of the decomposition and reduction, the contrast reaction without NaOH in different reaction times was carried out (Fig. S1). The results show that the reaction solution is still a colorless transparent solution and there were no obvious absorption peaks appeared in the time range of 3 to 60 min, implying that the silver bromide precipitate in this system do not decompose or the decomposition rate of silver bromide is negligible under light. For our normal reaction for the formation of AgNPs by adding NaOH, only 3 min was needed to carry out the synthesis of silver triangular nanoplates (Fig. S1 (A)) and nanorods or near nanospheres (Fig. S1 (B)). As a result, the competition of the decomposition silver bromide has little influence on the formation of AgNPs in our reaction system.

2) The effects of natural light on the formation of silver seeds prepared without adding KBH_4_


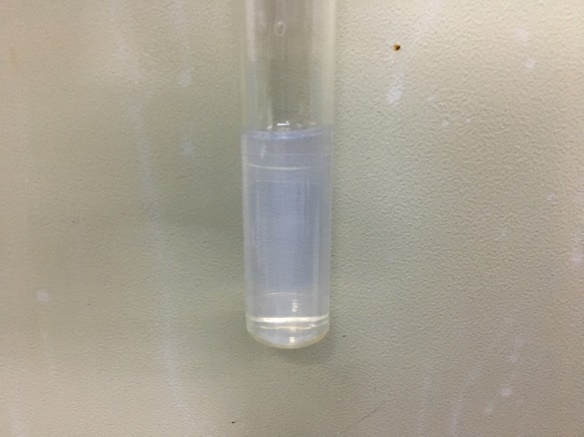


Fig. S2 UV-vis spectra of silver seeds prepared without KBH_4_ and aged at different times.

For the formation of silver seeds by adding CTAB and TSC but without KBH_4_ in our system, the color of the reaction solution is always milky white in 60 min and do not changes with the reaction time (Fig. S2). That is, the AgBr precipitate in the formation of silver seeds do not decompose to form AgNPs or the decomposition rate of AgBr is negligible under light.

2. The effect of unreacted BH_4_^-^ in the short aging time of seed solutions on the information of AgNPs

**C)**

**B)**

**A)**

**D)**

Fig. S3 UV-vis spectra of the silver seeds prepared by adding different quantity of BH_4_^-^ and corresponding AgNPs: silver seeds aged for 5 min (A) and 15 min (C); AgNPs prepared by the seeds aged for 5 min (B) and 15 min (D).

In order to verify the effect of unreacted BH_4_^-^ in the short aging time of seed solutions on the information of AgNPs, we carried out the above experiments by changing the addition amount of KBH_4_. In the samples of the seeds aged for 5 min (Fig. S3 (A) (B)) and 15 min ((C) (D)), the increase or decrease in the KBH_4_ has no obviously changes in the characteristic absorption peaks of both silver seeds and AgNPs at different aging times of silver seeds. The results imply that unreacted BH_4_^-^ have little influence on the formation of silver seeds and AgNPs prepared by the seeds. That is to say, it is not a key factor to determine the morphologies of the formed nanoparticles. The experiment by using the seeds aged for 30 min has the similar results.

3. The low magnification TEM images of silver nanorods and triangular nanoplates


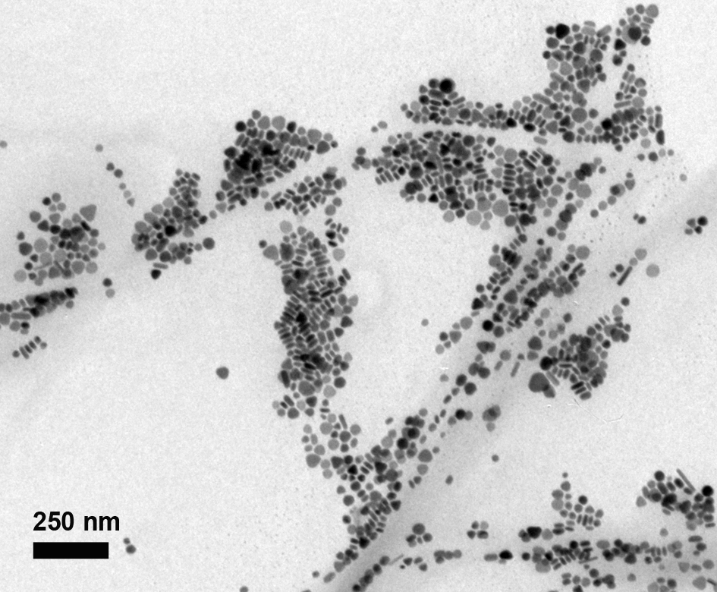

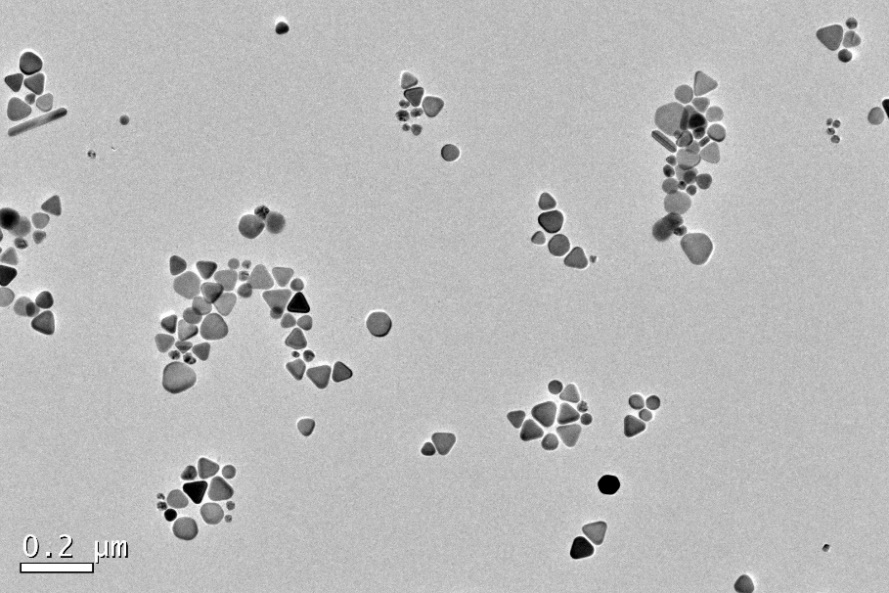


Fig. S4 Low magnification TEM images of silver nanorods (left) and triangular nanoplates (right) corresponding to Fig.1(C) and (E).

Some aggregated and irregular nanoparticles were mixed with silver nanorods or triangular nanoplates. Thus, it is difficult to determine the size distribution of these hybrid nanoparticles. The shape-distribution histograms of AgNPs corresponding to the TEM images of Fig. 1(C) and (E) were presented in Fig. 1(D) and (F), we did not consider to determine the shape-distribution corresponding to Fig. S4.
